# Development and validation of a prediction model on severe maternal outcomes among pregnant women with pre-eclampsia: a 10-year cohort study

**DOI:** 10.1038/s41598-020-72527-0

**Published:** 2020-09-24

**Authors:** Jing Tan, Min Yang, Yuan Liao, Yana Qi, Yan Ren, Chunrong Liu, Shiyao Huang, Lehana Thabane, Xinghui Liu, Xin Sun

**Affiliations:** 1 Chinese Evidence-Based Medicine Center, West China Hospital, Sichuan University, Chengdu, China; 2 Department of Health Research Methods, Evidence, and Impact, McMaster University, Hamilton, ON Canada; 3 Biostatistics Unit, St Joseph’s Healthcare Hamilton, Hamilton, ON Canada; 4 West China School of Public Health, Sichuan University, Chengdu, 610041 Sichuan People’s Republic of China; 5 West China Research Center for Rural Health Development, Sichuan University, Chengdu, Sichuan People’s Republic of China; 6 School of Medicine, University of Nottingham, Nottingham, UK; 7 Department of Obstetrics and Gynecology, and Key Laboratory of Birth Defects and Related Diseases of Women and Children (Sichuan University), Ministry of Education, West China Second University Hospital, Sichuan University, Chengdu, China

**Keywords:** Diseases, Health care, Risk factors

## Abstract

Pre-eclampsia is a severe hypertensive disorder of pregnancy and could lead to severe maternal morbidities and death. Our study aimed to develop and validate a prognostic prediction model for severe maternal outcomes among Chinese population with pre-eclampsia. We conducted a 10-year cohort study in a referral center by collecting all pregnant women who diagnosed as pre-eclampsia and delivered from 2005 to 2014. A composite of severe maternal outcomes, including maternal near-miss defined by World Health Organization, cortical blindness/retinal detachment, temporary facial paralysis and maternal death, were adopted. We used logistic regression model to develop Model 1 by retaining the predictors of *p* < 0.05, and further conducted Model 2 by adding quadratic terms and interaction terms to Model 1. We undertook a bootstrapping validation and estimated the model performance. A total of 397 pregnant women suffered from severe maternal outcomes among 2,793 eligible participants, with an incidence of 14.21% (95% confidence interval (CI) 12.91%–15.51%). Of 13 predictors were finally selected in Model 1. Combined with quadratic and interactive terms, the Model 2 showed higher area under the ROC curve (82.2%, 95% CI 79.6%–84.7%) and good calibration. By the bootstrapping validation, similar model performances were present.

## Introduction

Pre-eclampsia is a severe hypertensive disorder of pregnancy that is characterized by the presence of de novo hypertension after 20 weeks’ gestation. It accompanied with proteinuria and/or maternal organ dysfunctions, including acute kidney injury, liver dysfunction, hemolysis or thrombocytopenia, neurological features, and fetal growth restriction^1^. As the main cause of severe maternal morbidities and death, pre-eclampsia results in approximately 50,000 to 60,000 maternal deaths per year worldwide^2,3^, accounting for approximately 10% to 15% of maternal deaths^4^. The incidence of pre-eclampsia varies greatly among different countries and regions. Previous studies have shown that the global incidence of pre-eclampsia in 2013 was 4.6%^5^ and 2% to 6% in China ^6^. However, mainstay of deaths from pre-eclampsia occur in developing countries ^7,8^.

The pathogenesis of pre-eclampsia is still unclear^9^. However, part of pre-eclampsia could occur severe maternal and perinatal adverse outcomes before or after delivery, such as liver rupture, stroke, kidney failure, coagulation abnormalities, postpartum hemorrhage, even maternal death and stillbirth^10,11^. As a result, high-risk pregnant women with pre-eclampsia should be identified as early as possible and occurrence of severe adverse outcomes needs to be reduced through early intervention and timely termination of pregnancy. Previous studies have shown that the opinions of experts may not be accurate in predicting the occurrence of severe adverse outcomes^12^. Accordingly, there were a few prediction models were developed to foresee the severe maternal outcomes. However, these studies were conducted in developed countries, including mostly Caucasian populations with relatively few East Asian subjects. In light of different pathogenesis and risk factors of pre-eclampsia reporting in different population^13,14^, there is little known of the effect regarding these differences on prognosis of pre-eclampsia^15^. Additionally, the performance of the prediction model depends to a large extent on appropriate selection and measurement of the specific characteristics among target population and setting^16,17^; hence, considering the candidate predictors of the localized population and the corresponding medical system was a basis for developing prediction model.

In 2016, China fully implemented the Two-child policy, which significantly increased the proportion of older pregnant women^18^ and the corresponding incidence of pre-eclampsia^19^. As a result of the huge gap of quantity and quality on medical resources in different areas of China, early prediction tools are urgently required by junior clinicians and primary institutions, in order to screen high-risk pregnant women and further to timely achieve referral. Therefore, we developed and validated a prediction model for severe maternal outcomes in pregnant women with pre-eclampsia, using a 10-year cohort data ranged from 2005 to 2014.

## Materials and methods

This study was reported in accordance with the Transparent Reporting of a Multivariable Prediction Model for Individual Prognosis or Diagnosis (TRIPOD) statement.

### Ethic statement

The retrospective cohort study was designed according to the guidelines of the Declaration of Helsinki. Since this study involved the retrospective extraction and review of existing data, specific informed consent was exempted by the Medical Ethics Committee of West China Second Hospital of Sichuan University and the Institutional Review Board (IRB). The study protocol was approved by IRB (2013-003).

### Study design

We conducted a retrospectively 10-year cohort study in West China Second Hospital of Sichuan University, which is a teaching hospital and referral center for maternal and neonatal care in Western China. By collecting the data of all pregnant women who diagnosed as pre-eclampsia and delivered at West China Second Hospital from May 1, 2005 to December 31, 2014, we developed and validated a prediction model for severe maternal outcomes in pregnant women with pre-eclampsia. The intended timing of using this model is when a pregnant woman has been diagnosed with pre-eclampsia and one would be interested in the risk of a woman for developing serious adverse outcome. The span of time for prediction was from the diagnosis of pre-eclampsia to delivery and discharge within seven days after diagnosis. We used a pre-designed and tested case report form (CRF) to collect data from the medical records by chart review.

### Data collection

Prior to data collection, we designed and tested the structured CRF by combining with published literatures and experts’ opinions, and the established data extraction and input criteria, including definition and source of each variable, data input process and data check requirement. A team composed of postgraduate students and nurses trained in obstetrics were recruited as investigators. Before data extraction, all the researchers who were involved in data extraction were properly trained. Data extraction and input were completed by investigators through double input, and a consistency check was conducted afterwards. The data were locked after verification.

### Inclusion and exclusion criteria

We included pregnant women who delivered in the West China Second Hospital of Sichuan University from May 1, 2005 to December 31, 2014, who were eligible with either of the following conditions. (1) Pregnant women were diagnosed with pre-eclampsia on admission. (2) Pregnant women were not diagnosed with pre-eclampsia, but reached the diagnostic criteria in the 2013 guideline of Hypertension in Pregnancy by the American College of Obstetricians and Gynecologists (ACOG) on admission^20^. We excluded pregnant women who transferred to other hospitals after pre-eclampsia diagnosis.

### Study outcome

To comprehensively consider multiple organ dysfunction caused by pre-eclampsia, this study adopted a composite of severe maternal outcomes in pregnant women, including maternal near-miss and maternal death. According to the definition of the World Health Organization (WHO)^21^, maternal near-miss consists of a series of organ dysfunction, including respiratory dysfunction, cardiovascular dysfunction, renal dysfunction, coagulation/blood dysfunction, liver function disorder, neurological dysfunction, and uterine dysfunction. In light of the specificity of pre-eclampsia, other two severe adverse outcomes, namely cortical blindness/retinal detachment and temporary facial paralysis (Bell’s palsy), were added in this study. All outcome indicators were presented in Table 1.

**Table 1:** The characteristics of included population.

Characteristics	Median (interquartile range)/number (percentage)
Maternal age (year)	31 (27–35)
Pre-pregnancy BMI (kg/m^2^)	22.30 (20.03–24.97)
Rural residents	1208 (43.9)
Gestational week	36 (33–38)
Systolic pressure at admission (mmHg)	147 (134–163)
Diastolic pressure at admission (mmHg)	94 (85–105)
Number of gravities	2 (1–4)
Multipara	1031 (36.91)
Use of ART	182 (6.53)
Multiple gestations	386 (13.88)
Placenta previa	121 (4.33)
Oligohydramnios	148 (5.30)
History of cesarean section	404 (14.49)
History of gestational hypertension	98 (3.51)
History of stillbirth	71 (2.54)
Intrahepatic cholestasis	429 (15.36)
Hypertension	209 (7.48)
Diabetes mellitus	126 (4.51)
Gestational diabetes mellitus	424 (15.18)
HBsAg positivity	191 (6.84)
Cardiac diseases	73 (2.61)
IDA	295 (10.56)
Thalassemia	12 (0.43)
Neurological and mental diseases	17 (0.61)
Chronic nephritis	57 (2.04)
Other urinary system diseases	16 (0.57)
Immune system diseases	48 (1.72)
Hyperthyroidism	35 (1.25)
Hypothyroidism	88 (3.15)
Fatty liver	16 (0.57)
Hypoproteinemia	155 (5.55)

*BMI* body mass index, *ART* assisted reproductive technology, *HBsAg* hepatitis B virus surface antigen, *IDA* iron deficiency anemia.

### Candidate predictors

In this study, based on previous literatures and accessibility of indicators in clinical practice, candidate predictors were considered from the following six aspects: demographic characteristics (maternal age (years), registered residential place, height (meter), pre-pregnancy weight (kilogram), and pre-pregnancy body mass index (BMI, kg/m^2^)), gestational characteristics (gestational age at admission (weeks), gestational age at delivery (weeks), gravidity, parity, use of assisted reproductive technology (ART), number of fetuses, placenta previa, and oligohydramnios), history of gestations (history of cesarean section, history of gestational hypertension, and history of stillbirth), admission diagnosis of gestational comorbidities and complications (intrahepatic cholestasis, diabetes mellitus (Type I or Type II), gestational diabetes mellitus (GDM), hepatitis B virus surface antigen (HBsAg) positivity, cardiac diseases, iron deficiency anemia (IDA), thalassemia, neurological and mental diseases, chronic nephritis, other urinary system diseases, immune system diseases, hyperthyroidism, hypothyroidism or subclinical hypothyroidism, fatty liver and hypoproteinemia (serum albumin was less than 25 g)), symptoms at admission (edema, chest pain, dyspnea, nausea and vomiting, dizziness and headache, blurred vision, and itchy skin), and laboratory tests at admission (systolic blood pressure (mmHg), diastolic blood pressure (mmHg), platelet count (10^9^/L), fibrinogen (mg/dL), alanine transferase (U/L), aspartate transferase (U/L), total bilirubin (mmol/L), urea nitrogen (mmol/L), creatinine (mmol/L), and proteinuria). Among them, symptoms and laboratory tests were measured at admission in pregnant women with pre-eclampsia.

Regarding to the classification and definition of potentially candidate predictors, the registered residential place was divided into city and rural area. The pre-pregnancy BMI was calculated as pre-pregnancy body weight divided by square of height. The parity was divided into multipara and nullipara. The number of fetuses was divided into singleton and multiple gestations. Placenta previa was defined as a placenta that covered or was close to the cervix, and diagnosed by prenatal ultrasound. Oligohydramnios was defined as an amniotic fluid index with less than five centimeter or maximum depth of the amniotic pool with less than 2 cm as diagnosed by ultrasound. Chronic nephritis included diabetic nephropathy, nephrotic syndrome, immunoglobulin A nephropathy, hypertensive nephropathy, and lupus nephropathy. Other urinary system diseases included hydronephrosis and kidney stones. Cardiac diseases included congenital heart disease, rheumatic heart disease, and cardiomyopathy. Neurological and psychiatric disorders included any type of epilepsy, depression, and anxiety. Immune system diseases included systemic lupus erythematosus and antiphospholipid syndrome. Qualitative proteinuria at admission was graded as −, + , ++, +++ and ++++.

### Development and validation of the model

First, the association between each candidate predictor and the target outcomes was analyzed by univariable analysis. The significance test of candidate predictors with *p* < 0.1 were used for further analysis. Second, a correlation matrix of all of the candidate predictors with *p* < 0.1 was then established. If the correlation coefficient between variables was more than 0.8, then combined with clinical judgment, one of the variables was selected for the model, and the other highly correlated variable was abandoned. Furthermore, according to the missing ratio of the original data, no further treatment was conducted for variables with smaller than 5% of missing values. For predictors with a missing ratio more than 5%, the data were imputed using multivariate imputation by chained equations (MICE), and the iterations were set to 10 times. Finally, the Rubin’s rules were then used to combine the estimation results by logistic regression model ^22^. Variables were used as predictors if the *p*-value of partial regression coefficient is less than 0.05. We develop Model 1 based on above progress.

Next, a nonlinear term (quadratic term) was considered for all quantitative variables selected by above progress. We added the quadratic term of quantitative variables that were established in Model 1. If regression coefficients of quadratic term were statistically significant (*p* < 0.05), the linear and quadratic terms of the quantitative variable were included as predictors; otherwise, retained the linear term.

Additionally, on the basis of clinical judgment, possible interactions between the included predictors were considered. If the regression coefficient of the interaction term was statistically significant (*p* < 0.05), the interaction term was included in the prediction model; otherwise, the interaction term was abandoned. We finally conducted Model 2 by adding quadratic terms and interaction terms to Model 1.

To assess the performance of prediction model, we conducted an internal validation with bootstrapping. After 400 times of bootstrapping, the performance and the optimistic value of the model was calculated.

### Performance of the model

We reported the *χ*^*2*^ statistics, pseudo R^2^, Pearson *χ*^*2*^ and Akaike Information Criterion (AIC) to evaluate the overall goodness-of-fit of model. The discrimination ability of the model was measured by the area under the receiver operating characteristics (ROC) curve; we also reported the sensitivity, specificity, predictive accuracy, positive predictive value, negative predictive value and the discriminant slope, respectively. The Hosmer–Lemeshow test was used to estimate the calibration ability of the model and also draw the calibration plot. Smoothing technology and the Loess algorithm were used to describe the relationship between the observed probability and the predicted probability of the outcomes. To screen the high-risk population as possible, the prediction model was considered to be more valuable when the sensitivity was > 70%. Therefore, an optimum cut-off value of the model was selected to achieve a sensitivity of > 70% and a prediction accuracy of > 70%. All statistical analyses were conducted by STATA 13.0 (StataCorp, College Station, TX, USA).

## Results

### Characteristics of the cohort

A total of 2,793 eligible pregnant women with pre-eclampsia who delivered between May 1, 2005 and December 31, 2014 were enrolled in this study. The number of participants per year ranged from 108 to 390. Among these, the median age was 30 years (interquartile range, 27–35 years). The median gestational age was 36 weeks (33–38 weeks). A total of 56.1% of the women were urban residents, 36.9% of them were multipara, and 13.9% had multiple pregnancies. The characteristics of included population were presented (Table 1). A total of 397 pregnant women with pre-eclampsia suffered from severe maternal outcomes, with an incidence of 14.21% (95%CI: 12.91–15.51%). The median time from diagnosis of pre-eclampsia to delivery was 48 h (interquartile range, 24–120 h). The numbers of pregnant women with respiratory dysfunction, cardiovascular dysfunction, renal dysfunction, coagulation dysfunction, hepatic dysfunction, neurological dysfunction, uterine dysfunction, specific symptom and maternal death were 124, 113, 155, 233, 8, 31, 6, 13, and 5 respectively (Table 2).

**Table 2:** Numbers of severe maternal outcomes.

Category	Indicators	Number^a^
Respiratory dysfunction	Acute cyanosis	7
	Gasping	2
	Severe tachypnea or bradypnea (respiratory rate > 40 breaths per minute or < 6 breaths per minute)	10
	Severe hypoxemia (O_2_ saturation < 90% for ≥ 60 min or PAO_2_/FiO_2_ < 200)	41
	FiO_2_ ≥ 50% for 1 > hour	35
	Intubation and ventilation not related to anesthesia	29
Cardiovascular dysfunction	Shock	6
	Use of continuous vasoactive drugs	71
	Cardiac arrest	10
	Cardiopulmonary resuscitation	9
	Severe hypoperfusion (lactate > 5 mmol/l or > 45 mg/dl)	10
	Severe acidosis (pH < 7.1)	7
Renal dysfunction	Dialysis for acute renal failure	36
	Oliguria non-responsive to fluids or diuretics	6
	Severe acute azotemia (creatinine ≥ 300 µmol/ml or ≥ 3.5 mg/dl)	113
Coagulation/hematological dysfunction	Failure to form clots	48
	Massive transfusion of blood or red cells (≥ 5 units)	156
	Severe acute thrombocytopenia (< 50 000 platelets/ml)	29
Hepatic dysfunction	Jaundice	1
	Severe acute hyperbilirubinemia (bilirubin > 100 µmol/l or > 6.0 mg/dl)	7
Neurological dysfunction	Prolonged unconsciousness (lasting ≥ 12 h)/coma	23
	Stroke	0
	Epilepticus	8
Uterine dysfunction	Hysterectomy	6
Specific symptom	Cortical blindness/retinal detachment	10
	Temporary facial paralysis	3
Death	Maternal death	5
Total	–	397

^a^The subtotals of each indicator were overlapped.

### Selection of predictors

Normality tests were conducted for all quantitative variable; of those, the original data of hemoglobin, platelet, fibrinogen, alanine transferase, aspartate transferase, total bilirubin, urea nitrogen, and creatinine levels at admission with skewed distribution were included in our analysis after logarithmic transformation. Univariable analysis of the associations between candidate predictors and outcomes showed that there were 32 variables with *p* < 0.1, which included maternal age, pre-pregnancy BMI, registered residential place, gestational week at delivery, multiple gestations, use of ART, placenta previa, diabetes mellitus, GDM, HBsAg positivity, heart diseases, IDA, chronic glomerulonephritis, immune system diseases, fatty liver, hypoproteinemia, edema, chest pain, dyspnea, nausea and vomiting, dizziness and headache, blurred vision, admission systolic pressure, admission diastolic pressure, log-transformed platelets, log-transformed fibrinogen, log-transformed alanine transferase, log-transformed aspartate transferase, log-transformed total bilirubin, log-transformed urea nitrogen, log-transformed creatinine, and qualitative proteinuria (Table S1).

The correlation coefficient matrix analysis showed that log-transformed alanine transferase levels and log-transformed aspartate transferase levels were highly correlated, with a correlation coefficient of 0.914, the systolic blood pressure and diastolic blood pressure at admission with a correlation coefficient of 0.803, thus we retained log-transformed aspartate transferase levels and systolic blood pressure at admission in consideration of clinical values.

There were three predictors with missing data of more than 5%, including urinary protein (10.92%), urinary nitrogen (6.34%), and creatinine (5.66%) (Table S1). After multiple imputation, 2,507 individuals of data samples were obtained.

Of 13 predictors were selected by multivariable logistic regression using imputed data set (Table S2). These predictors were gestational age, placenta previa, HBsAg positivity, heart diseases, IDA, dyspnea, systolic blood pressure at admission, log-transformed platelets, log-transformed fibrinogen, log-transformed aspartate transferase, log-transformed total bilirubin, log-transformed creatinine, and qualitative proteinuria. Based on these 13 predictors, we developed the Model 1. After adding quadratic term and interaction term, we also presented the Model 2 (Table 3).

**Table 3:** Included predictors and corresponding odds ratios in models.

Variables	Model 1		Model 2	
	OR (95% CI)	Coefficient (95% CI)	OR (95% CI)	Coefficient (95% CI)
Constant	0.26 (0.008–8.71)	− 1.35 (− 4.86 to 2.16)	3.43e + 14	33.47 (22.16–44.78)
Gestational week	0.95 (0.92–0.98) **	− 0.05 (− 0.08 to − 0.01) **	0.95 (0.91–0.98) **	− 0.05 (− 0.09 to − 0.01) **
Placenta previa	2.56 (1.62–4.05) ***	0.94 (0.48–1.40) ***	2.81 (1.75–4.51) ***	1.03 (0.56–1.51) ***
HBsAg positivity	1.72 (1.11–2.67) *	0.54 (0.11–0.98) *	1.99 (1.28–3.10) **	0.69 (0.25–1.13) **
Cardiac diseases	3.28 (1.72–6.28) ***	1.19 (0.54–1.84) ***	3.70 (1.94–7.06) ***	1.31 (0.66–1.95) ***
IDA	2.93 (2.07–4.15) ***	1.08 (0.73–1.42) ***	2.89 (2.02–4.11) ***	1.06 (0.71–1.41) ***
Dyspnea	3.41 (2.02–5.76) ***	1.23 (0.70–1.75) ***	2.93 (1.73–4.97) ***	1.08 (0.55–1.60) ***
Systolic blood pressure	1.01 (1.00–1.01)	0.005 (− 0.0003 to 0.01)	1.01 (1.00–1.01) *	0.006 (0.0008–0.01) *
Platelet count^a^	0.45 (0.35–0.59) ***	− 0.79 (− 1.06 to 0.53) ***	6.29e − 06 (2.27e − 07–0.0002) ***	− 11.98 (− 15.30 to − 8.65) ***
Fibrinogen^a^	0.41 (0.28–0.60) ***	− 0.90 (− 1.29 to − 0.51) ***	0.38 (0.26–0.56) ***	− 0.96 (− 1.36 to − 0.57) ***
Aspartate transferase^a^	1.20 (1.01–1.43) *	0.18 (0.01–0.36) *	1.22 (1.02–1.45) *	0.20 (0.02–0.37) *
Total bilirubin^a^	1.53 (1.18–1.98) **	0.42 (0.17–0.68) **	1.40 (1.07–1.85) **	0.39 (0.12–0.66) **
Creatinine^a^	4.67 (3.36–6.48) ***	1.54 (1.21–1.87) ***	0.61 (0.11–3.35)	− 0.50 (− 2.21 to 1.21)
Urine protein				
+	2.78 (0.83–9.29)	1.02 (− 0.19 to 2.23)	2.58 (0.77–8.60)	0.95 (− 0.26 to 2.15)
+ +	2.79 (0.83–9.42)	1.03 (− 0.19 to 2.24)	2.52 (0.75–8.50)	0.93 (− 0.29 to 2.13)
+ + +	3.85 (1.14–12.98) *	1.35 (0.13–2.56) *	3.45 (1.03–11.60) *	1.24 (0.03–2.45) *
+ + + +	4.16 (1.24–13.88) *	1.42 (0.22–2.63) *	3.82 (1.15–12.71) *	1.34 (0.14–2.54) *
Nonlinear term of platelet^a^	–	–	2.30 (1.45–3.63) ***	0.83 (0.37–1.29) ***
Platelet × creatinine^a^	–	–	1.09 (1.02–1.17) *	0.08 (0.01–0.15) *

*IDA* iron deficiency anemia.

*P< 0.05, ** *P* < 0.01, ***P < 0.001.

^a^Logarithmic transformation.

### Model performance

The multiple indicators indicated that two logistic regression models had overall significance, with a well goodness-of-fit (*p* > 0.05). After inclusion of quadratic and interaction term, the pseudo *R*^*2*^ significantly increased and AIC value deceased (Table 4).

**Table 4:** The main indicators of model performance.

Performance	Indicators	Model 1	Model 2
**Overall performance**	Pseudo *R*^*2*^	0.224	0.258
	*χ*^*2*^ statistics	434.01 (*p * < 0.001)	500.70 (*p * < 0.001)
	Pearson *χ*^*2*^	2,271.64 (*p* = 0.29)	2,274.32 (*p* = 0.27)
	Akaike information criterion (AIC)	0.683	0.656
**Discrimination**	Area under ROC curve (95% CI)	80.62% (78.00–83.25%)	82.15% (79.60–84.70%)
	Cut-off	0.14	0.14
	Sensitivity	71.55%	72.70%
	Specificity	73.56%	76.13%
	Accuracy	73.25%	75.60%
	Positive predictive value	33.02%	35.68%
	Negative predictive value	93.42%	93.87%
	Discrimination slope	0.236	0.275
**Calibration**	Hosmer–Lemeshow goodness-of- fit *χ*^*2*^	8.95 (*p* = 0.35)	12.93 (*p* = 0.11)

*ROC* receiver operating characteristics curve, *CI* confidence interval.

**Figure 1:**
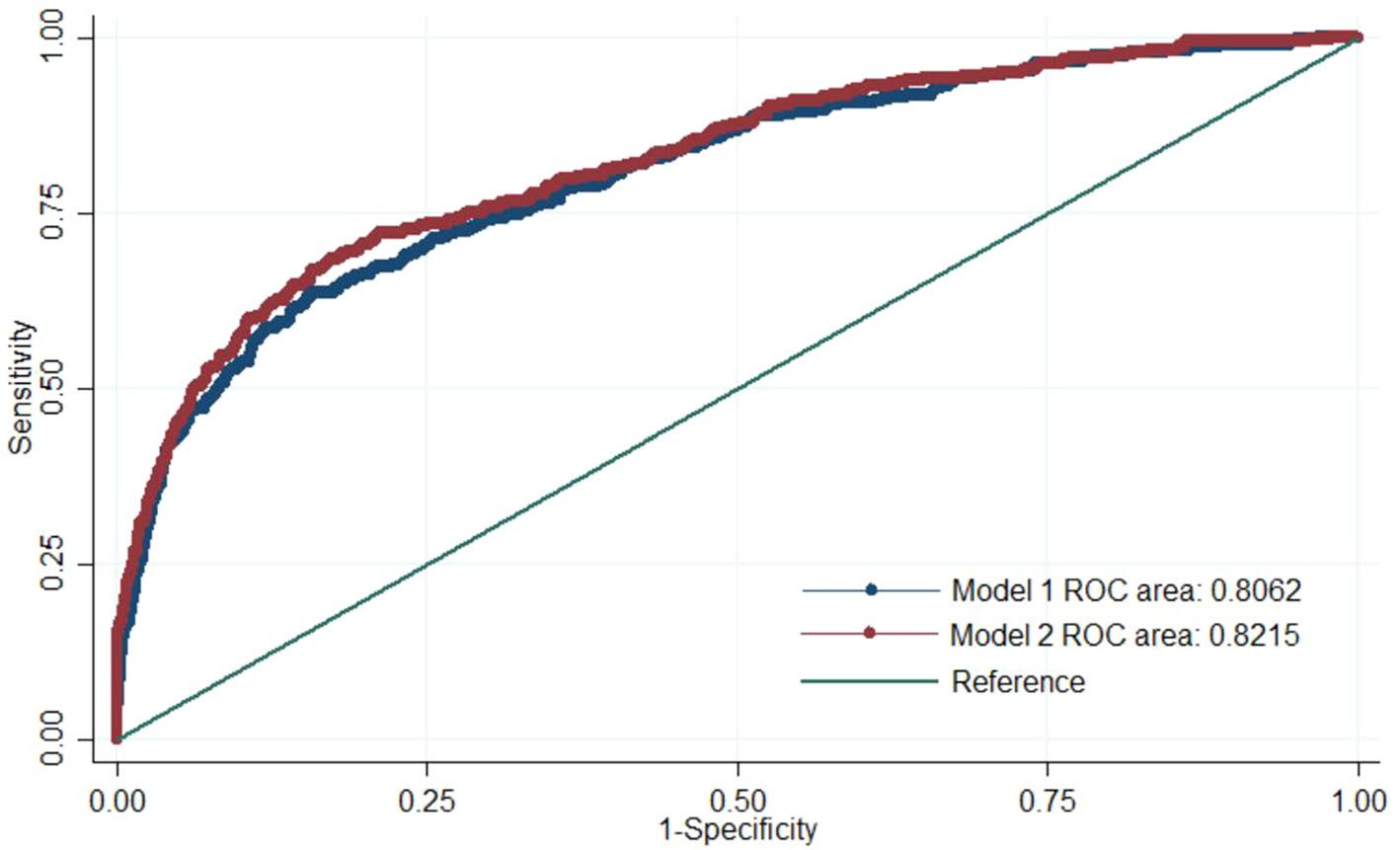
Area under the ROC curve. Blue curve represents the area of Model 1, and red curve represents the area of Model 2.

By adding quadratic and interactive terms, Model 2 showed the higher area under the ROC curve (82.2%, 95% CI 79.6–84.7%) (Fig. 1, Table 4). When we set the cut-off as 0.14 in consideration of the incidence of outcome, Model 2 had the higher sensitivity (72.7%), specificity (76.1%), predictive accuracy (75.6%), and discrimination slope (0.27) (Table 4).

**Figure 2:**
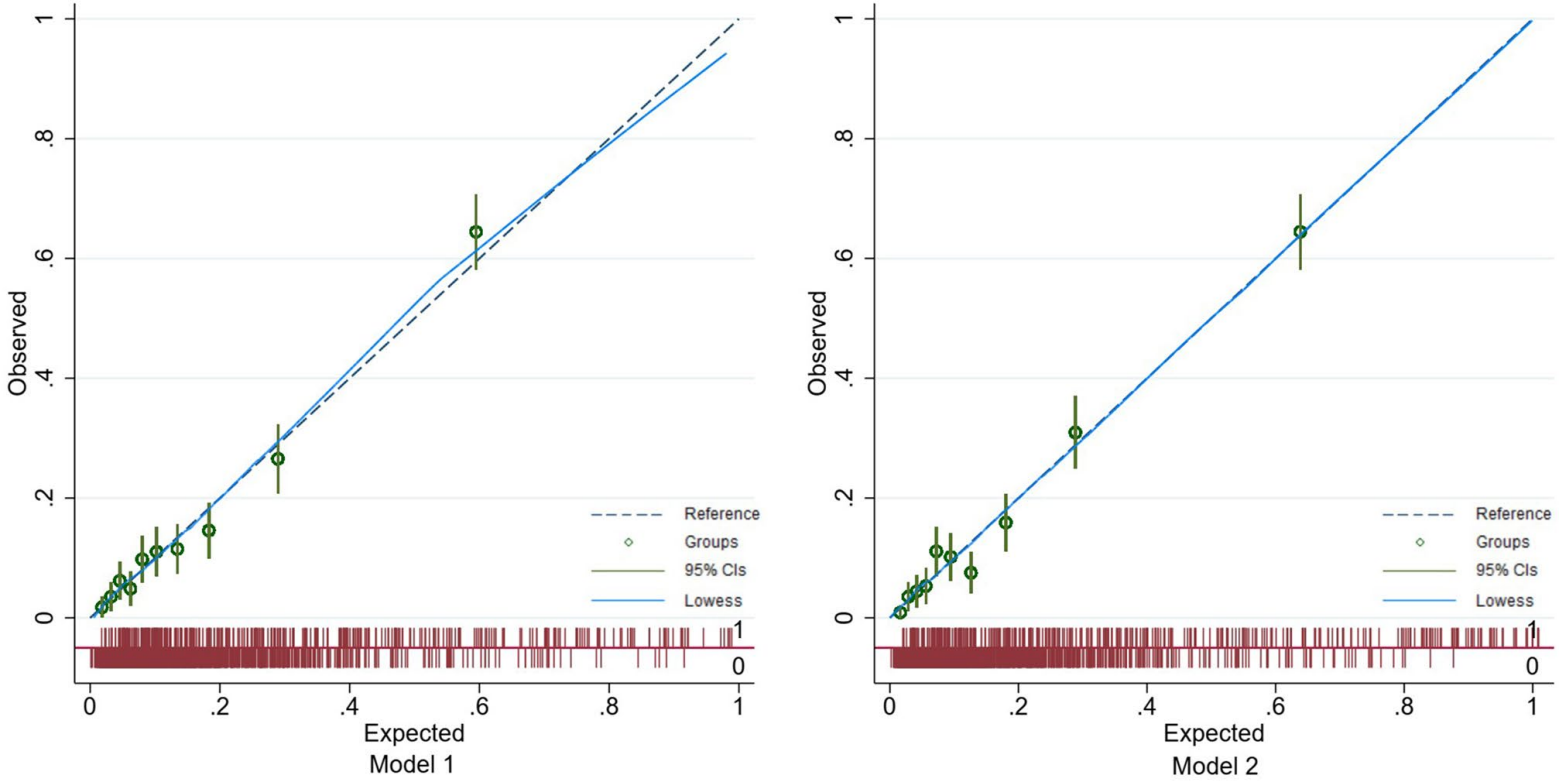
Calibration plots of Model 1 and Model 2. Blue dotted lines are the reference line, and blue curves are the display of the lowess smoother; green cycles mean predicted risks are divided into 10 equally sized groups and green lines mean the 95% confidence intervals for each group.

The Hosmer–Lemeshow tests in two models showed *p* > 0.05. Combing with calibration plot, we suggested that the calibration abilities of Model 2 was better than Model 1 (Fig. 2, Table 4). Therefore, Model 2 was finally selected as the prediction model of severe maternal outcomes in pregnant women with pre-eclampsia.

Internal validation by bootstrapping showed that the optimism of the prediction model was 0.004, which indicated that the model was not apparent over-fitting (Tables S3).

Given that severe acute azotemia and massive transfusion accounted for two-thirds of all composite outcomes, we conducted a sensitivity regardless of gestational age analysis by merely including these two subjective outcomes. We acquired the slightly better model performance of discrimination and calibration (Tables S4).

## Discussion

### Main findings

Using a 10-year cohort of 2,793 pregnant women who diagnosed as pre-eclampsia in West China Second University Hospital of Sichuan University from 2005 to 2014, we developed and validated internally a prediction model of severe maternal outcomes in pregnant women with pre-eclampsia , and got a good discrimination (e.g. 82.2% of area under the ROC curve). The model was presented as follows: logit (P) = 33.468 − 0.051 gestational week + 1.033 placenta previa + 0.690 HBsAg positivity + 1.308 cardiac diseases + 1.060 IDA + 1.075 dyspnea + 0.006 systolic blood pressure − 11.976 log-transformed platelets − 0.964 log-fibrinogen + 0.198 log-transformed aspartate transferase + 0.391 log-transformed bilirubin—0.497 log-transformed creatinine + 0.946 urine protein ( +) + 0.926 urine protein (++) + 1.239 urine protein (+++) + 1.340 urine protein (++++) + 0.832 log-transformed platelets^2^ + 0.085 log-transformed platelets × log-transformed creatinine. When 0.14 was used as the cut-off value, the sensitivity of the model was 72.70%, the specificity was 76.13%, the predictive accuracy was 75.60%, the positive predictive value was 35.68%, and the negative predictive value was 93.87%. The calibration ability of the model was also well.

### Comparison with previous studies

A few number of prediction models aiming at severe maternal outcomes among pregnant women with pre-eclampsia were developed before^9,23–26^. Of those, fullPIERS model^9^ and miniPIERS model^25^ were the main parts of PIERS study, while other two extended models were also reported (A miniPIERS model with SpO2^24^ and a combined cardiorespiratory symptom model^23^). Another recent PREP model was conducted in 2017 by enrolling 946 individuals in UK^26^. Compared with these models, our study firstly involved Asian population and fully consider the characteristics of Chinese pregnant women.

Second, we finally used 13 variables for predicting and got a similar model performance to previous models (e.g. area under ROC curve more than 0.80, sensitivity and specificity more than 0.70); of those, three predictors have not been considered in previous models, involving placenta previa, HBsAg positivity and IDA. It emphasized the specific characteristics and heterogeneous baseline-risk for Chinese population, and more attention should be paid for these subgroups. We further discussed the implications of three predictors in later paragraph. Specially, all the predictors in our model are easily available across Chinese setting; however, some predictors such as SpO2, has significant predictive ability in PIERS model, whereas it is difficult to universally acquire in Chinese setting, and also had more than 50% missing values in PREP’s study.

Third, our study included all kind of pregnant women diagnosed with pre-eclampsia, whereas PREP model only included the pregnant women with early-onset pre-eclampsia diagnosed before 34 weeks gestation.

### Included predictors

Based on the literature and availability of indicators in clinical practice, 48 candidate predictors from six aspects (demographic and sociological characteristics, basic characteristics of pregnancy, pregnancy and childbirth history, admission diagnosis, admission symptoms, and laboratory tests) were considered in this study. A total of 13 variables were included in the final optimal logistic regression model as predictors. We found that gestational age at admission was a predictor of severe adverse outcomes. Pregnant women who had a lower gestational age at admission appeared to have worse severe adverse outcomes compared with pregnant women with a higher gestational age. Pre-eclampsia can be divided into two types according to the difference in occurrence time: early-onset pre-eclampsia and late-onset pre-eclampsia. Early-onset pre-eclampsia usually occurs before 34 weeks of gestation, while late-onset pre-eclampsia occurs after 34 weeks of gestation^27,28^. Previous studies have shown that there are significant differences in etiology and prognosis between the two groups, and early-onset pre-eclampsia has worse maternal and perinatal outcomes than late-onset pre-eclampsia^28^. Therefore, the predictor of gestational age at admission in our study indicates the admission time of pregnant women after diagnosis of pre-eclampsia, rather than encouraging pregnant women to delay admission. Timely diagnosis and treatments were beneficial to the prognosis.

Placenta previa, HBsAg positivity and IDA were firstly included in homogeneous prediction models. Placenta previa has always been deemed as an important predictor for severe adverse outcomes^29^. Several published studies have shown that there might be an interaction between pre-eclampsia and placenta previa. The incidence of placenta previa in women with pre-eclampsia was significantly lower than that in pregnant women without pre-eclampsia^30,31^, and the reverse is also true^32^. Although the mechanism of this interaction is still unclear, both pre-eclampsia and placenta previa are associated with abnormal infiltration of villous trophoblasts. Shallow trophoblast infiltration in women with pre-eclampsia (shallow implantation) leads to a decrease in placental perfusion, whereas deep trophoblast infiltration in pregnant women with placenta previa (deep implantation) leads to an increase in placental perfusion. Interaction of these two factors may reduce the risk of placenta previa to a certain extent^33^. With the above-mentioned mechanism, some pregnant women with pre-eclampsia still have placenta previa, thus severe adverse outcomes in these patients manifest as the combined effect of placenta previa and pre-eclampsia. This may significantly increase the risk of placental abruption, postpartum hemorrhage, hysterectomy, and other adverse outcomes^33^. In condition of the high cesarean section rate in China^34^ and the increased risk of placenta previa in the subsequent pregnancy after cesarean section delivery^35^, we suspected placenta previa was the main reason for massive blood transfusion. It accounted for the large proportion of composite outcomes.

Besides, cardiac diseases, HBsAg positivity and IDA had a significant effect on outcome. The main reason for this finding is that these gestational comorbidities may aggravate damage to the heart, liver, and circulation system of pregnant women. Patients with pre-eclampsia commonly occur high total vascular resistance index, partly mediated by a substantial increase in sympathetic vasoconstrictor activity^36^, which could increase the burden of heart. On the other hand, the cardiac output and stroke volume increase from the early pregnancy to delivery, thus those women with cardiovascular compromise were more likely to occur pulmonary edema during the second stage of labor and postpartum period^37,38^. The risk of severe adverse outcomes is then significantly increased.

The previous study reported that nearly 3% of pregnancies are complicated by a variety of liver disease, which can have fatal consequences for pregnant women and offspring^39^. More notably, Hepatitis B is high-endemic in China. A meta-analysis estimated that approximately 7.6% of pregnant women were affected^40^. Although the association between hepatitis B virus infection and pre-eclampsia is still uncertain^41–43^, hepatitis B virus carrier and replication may aggravate the abnormalities of liver function in pregnancy and pre-eclampsia that occur coincidentally. To our knowledge, the predictive ability of HBsAg positivity on severe maternal outcomes among pre-eclampsia women is firstly reported. With regard to the relatively higher prevalence of hepatitis B in Asia Pacific and sub-Saharan African^44^, the effects could not be ignored in similar population.

In our study, pre-eclampsia was defined as a composite of such disorders as maternal acute kidney injury, liver dysfunction, neurological features, hemolysis or thrombocytopenia. For this condition, several laboratory markers, such as platelets and creatinine, are important prognostic factors of the disease. The outcome of our interest is a composite of severe maternal outcomes that composes of multiple organ dysfunctions. One would observe that some laboratory markers measured at baseline were used for predicting the occurrence of the composite outcome which included a same variable given a predefined level (e.g. creatinine over 300). Although it is obvious that the laboratory markers at baseline may well predict their level in the future, inclusion of such laboratory markers as predictors would also help predict the occurrence of other outcomes. For instance, systolic blood pressure and proteinuria are core indicators for diagnosis and prognosis of pre-eclampsia^45,46^. Our study suggested that increased systolic blood pressure may lead to worse outcomes. Proteinuria is an important indicator of renal dysfunction in pre-eclampsia, but in raw data of this study, we found that more than 20% of pregnant women received no quantitative proteinuria measurement. Therefore, in the current study, qualitative analysis of proteinuria was used as a candidate predictor instead of quantitative detection. In future studies, our team will further determine the effect of quantitative proteinuria to further optimize the model. Other predictors, including log-transformed platelets, log-fibrinogen, log-transformed aspartate transferase, log-transformed bilirubin, and log-transformed creatinine are the main indicators of functional status of vital organs. This is consistent with the mechanism of important organ damage caused by pre-eclampsia.

In addition, we included quadratic and interaction terms in Model 2. The choice of quadratic term and interaction term in prediction model was mainly driven by model performance, and the clinical significance of these mathematically sophisticated terms may be less likely uninterpretable. As such, we presented two models in our study. Model 1 did not include the quadratic and interaction terms, but model 2 did. In addition, these two models require different computational capacities. Clinicians may choose the one that work best for their own purposes.

### Implication of our model

Our model provided a plausible predictive tool for identifying the high-risk pregnant women diagnosed pre-eclampsia among Chinese population. When a pregnant woman was diagnosed with pre-eclampsia, this model may offer a probability of severe maternal outcome which would assist clinician to determine whether the pregnant women need to be hospitalized and monitored more closely, as well as the decision-making about timing of delivery. This is particularly the case for junior clinicians and midwives in primary healthcare institutions. Given that the medical resources and healthcare capability are heterogenous among Chinese medical institutions, timely transferal for pre-eclampsia women at high risk might be an effective response to reduce the risk of severe maternal outcomes. Our model showed that setting a cut-off of 0.14 as the predicted probability presented a relatively good predictive ability (e.g. the sensitivity was 72.70% and the specificity was 76.13%). Note that this threshold in our model might be more useful in similar setting to our hospital. Further external validation and recalibration of this model are warranted before clinical use in different medical environment and broader population.

### Strengths and limitations of the study

This study has several strengths. First, to the best of our knowledge, this study is the first prediction model aiming at severe maternal outcomes in women with pre-eclampsia based on the Asian population; by collecting data from a regional maternal referral center over 10 years (2005–2014), we presents the largest sample size among similar studies. Second, it emphasized the accessibility of all predictors across Chinese setting and identified three gestational comorbidities without inclusion in previous studies, involving placenta previa, HBsAg positivity and IDA. Third, we have got a good discrimination and calibration by a strict data collection process and transparently statistical approach for model development and internal validation.

Our study also has the following limitations. First, this study was a retrospective cohort study. As result of comparatively lower incidence of pre-eclampsia among pregnant women, collecting a sufficient number of subjects in a short period of time is difficult if prospective studies are carried out. Therefore, a retrospective design was adopted in our study. Second, according to the definition of maternal severe outcomes by WHO, some subjective symptoms were included in the composite outcome, such as cyanosis, gasping, and jaundice. Although we used a pre-designed CRF to extract those from the medical records by chart review, the accuracy of symptoms is subject to the judgment and records by clinicians. Third, the current study just conducted development of the model and internal validation. External validation in other medical institutions will be conducted in the next stage, and research results will then be reported.

## Conclusions

In this study, a prediction model for severe maternal outcomes in pregnant women with pre-eclampsia was established using a multivariable logistic regression model. This model has a good predictive ability by internal validation. Further external validation is required to clarify the clinical applicability of our model.

## Supplementary information

Supplementary Information.

## Data Availability

Data sharing is possible upon ethnical approval and mutual agreement on authorship. Both the Chinese Evidence-based Medicine Center and West China Second Hospital are jointly responsible for granting the use of the study data.
