# Supplementary material for: Development and validation of a prediction model on severe maternal outcomes among pregnant women with pre-eclampsia: a 10-year cohort study
Source: Sci Rep. 2020 Sep 24;10:15590. doi: 10.1038/s41598-020-72527-0 (PMC7518280; doi:10.1038/s41598-020-72527-0)
Supplement: Supplementary file 1 — Supplementary Information. [file 41598_2020_72527_MOESM1_ESM.docx]

**Supplementary Tables**

**Development and validation of a prediction model on severe maternal outcomes among pregnant women with pre-eclampsia: a 10-year cohort study**

Jing Tan^1, 2^, Min Yang^3,4,5^, Yuan Liao^6^, Yana Qi^1^, Yan Ren^1^, Chunrong Liu^1^, Shiyao Huang^1^, Lehana Thabane^2^, Xinghui Liu^6*^, Xin Sun^1*^

^1^ Chinese Evidence-based Medicine Center, West China Hospital, Sichuan University, Chengdu, China

^2^ Department of Health Research Methods, Evidence, and Impact, McMaster University, Hamilton ON, Canada; Biostatistics Unit, St Joseph’s Healthcare—Hamilton, Hamilton ON, Canada

^3^ West China School of Public Health, Sichuan University, Chengdu, 610041, Sichuan, People's Republic of China

^4^ West China Research Center for Rural Health Development, Sichuan University, Chengdu, Sichuan, People's Republic of China.

^5^ School of Medicine, University of Nottingham, Nottingham, UK.

^6^ Department of Obstetrics and Gynecology, and Key Laboratory of Birth Defects and Related Diseases of Women and Children (Sichuan University), Ministry of Education; West China Second University Hospital, Sichuan University, Chengdu, China

**Corresponding authors**

^*^Xin Sun, [sunx79@hotmail.com](mailto:sunx79@hotmail.com); ^*^Xinghui Liu, [xinghuiliu@163.com](mailto:xinghuiliu@163.com)

**Table S1 Univariable analysis of the associations between candidate predictors and outcomes**

| **Candidate predictors** | **Number** | **Missing values (Number, (%))** | **Category** | **Women with outcome (Number, (%))** | **Women without outcome (Number, (%))** | ***P* value** |
| --- | --- | --- | --- | --- | --- | --- |
| **Demographic characteristics** | |  |  |  |  |  |
| Maternal age | 2790 | 3 (0.1) | — | 30.2 (6.4)^1^ | 30.9 (5.7)^1^ | **0.015** |
| Pre-pregnancy BMI | 2700 | 93 (3.3) | — | 22.2 (3.3)^1^ | 22.9 (4.2)^1^ | **0.023** |
| Registered residential place | 2752 | 41 (1.5) | City | 189 (48.0) | 1355 (57.5) | **<0.001** |
|  |  |  | Rural area | 205 (52.0) | 1003 (42.5) |  |
| **Gestational characteristics** |  |  |  |  |  |  |
| Gestational age at admission | 2765 | 28 (1.0) | — | 33.3 (4.0)^1^ | 34.8 (3.7)^1^ | **<0.001** |
| Gravidity | 2793 | 0 (0) | — | 2 (1-4)^2^ | 2 (1-4) ^2^ | 0.47 |
| Parity | 2793 | 0 (0) | Nullipara | 221 (55.7) | 1541 (64.3) |  |
|  |  |  | Multipara | 176 (44.3) | 855 (35.7) | **<0.001** |
| Use of ART | 2787 | 6 (0.2) | No | 381 (95.5) | 2224 (93.0) |  |
|  |  |  | Yes | 16 (4.5) | 166 (7.0) | **0.029** |
| Multiple gestations | 2780 | 13 (0.5) | No | 328 (84.3) | 2066 (86.4) |  |
|  |  |  | Yes | 61 (15.7) | 325 (13.6) | 0.27 |
| Placenta previa | 2793 | 0 (0) | No | 357 (89.9) | 2289 (95.5) |  |
|  |  |  | Yes | 40 (10.1) | 107 (4.5) | **<0.001** |
| Oligohydramnios | 2793 | 0 (0) | No | 380 (95.7) | 2265 (94.5) |  |
|  |  |  | Yes | 17 (4.3) | 131 (5.5) | 0.33 |
| **History of gestations** |  |  |  |  |  |  |
| History of cesarean section | 2788 | 5 (0.2) | No | 334 (84.3) | 2050 (85.7) |  |
|  |  |  | Yes | 62 (15.7) | 342 (14.3) | 0.48 |
| History of gestational hypertension | 2793 | 0 (0) | No | 384 (96.7) | 2311 (96.5) |  |
|  |  |  | Yes | 13 (3.3) | 85 (3.5) | 0.78 |
| History of stillbirth | 2793 | 0 (0) | No | 389 (98.0) | 2333 (97.4) |  |
|  |  |  | Yes | 8 (2.0) | 63 (2.6) | 0.47 |
| **Gestational comorbidities and complication** | | | |  |  |  |
| Intrahepatic cholestasis | 2793 | 0 (0) | No | 332 (83.6) | 2032 (84.8) |  |
| Hypertension | 2793 | 0 (0) | Yes  No  Yes | 65 (16.4)  360 (90.7)  37 (9.32) | 364 (15.2)  2224 (92.8)  117 (7.2) | 0.55  0.13 |
| Diabetes mellitus | 2793 | 0 (0) | No | 388 (97.7) | 2279 (95.1) |  |
|  |  |  | Yes | 9 (2.3) | 117 (4.9) | **0.020** |
| GDM | 2793 | 0 (0) | No | 358 (90.2) | 2011 (83.9) |  |
|  |  |  | Yes | 39 (9.8) | 385 (16.1) | **<0.001** |
| HBsAg positivity | 2793 | 0 (0) | No | 359 (90.4) | 2243 (93.6) |  |
|  |  |  | Yes | 38 (9.6) | 153 (6.4) | **0.020** |
| Cardiac diseases | 2793 | 0 (0) | No | 367 (92.4) | 2353 (98.2) |  |
|  |  |  | No | 30 (7.6) | 43 (1.8) | **<0.001** |
| IDA | 2793 | 0 (0) | No | 298 (75.1) | 2200 (91.8) | **<0.001** |
|  |  |  | No | 99 (24.9) | 196 (8.2) |  |
| Thalassemia | 2793 | 0 (0) | No | 396 (99.8) | 2385 (99.5) |  |
|  |  |  | Yes | 1 (0.2) | 11 (0.5) | 0.56 |
| Neurological and mental diseases | 2793 | 0 (0) | No | 394 (99.2) | 2382 (99.4) |  |
|  |  |  | Yes | 3 (0.8) | 14 (0.6) | 0.68 |
| Chronic nephritis | 2793 | 0 (0) | No | 380 (95.7) | 2356 (98.3) |  |
|  |  |  | Yes | 17 (4.3) | 40 (1.7) | **<0.001** |
| Other urinary system diseases | 2793 | 0 (0) | No | 396 (99.7) | 2381 (99.4) |  |
|  |  |  | Yes | 1 (0.3) | 15 (0.6) | 0.36 |
| Immune system diseases | 2793 | 0 (0) | No | 383 (96.5) | 2362 (98.6) |  |
|  |  |  | Yes | 14 (3.5) | 34 (1.4) | **0.003** |
| Hyperthyroidism | 2793 | 0 (0) | No | 389 (98.0) | 2369 (98.9) |  |
|  |  |  | Yes | 8 (2.0) | 27 (1.1) | 0.14 |
| Hypothyroidism or subclinical hypothyroidism | 2793 | 0 (0) | No | 383 (96.5) | 2322 (96.9) |  |
|  |  |  | Yes | 14 (3.5) | 74 (3.1) | 0.64 |
| Fatty liver | 2793 | 0 (0) | No | 389 (98.0) | 2388 (99.7) |  |
|  |  |  | Yes | 8 (2.0) | 8 (0.3) | **<0.001** |
| Hypoproteinemia | 2793 | 0 (0) | No | 354 (89.2) | 2284 (95.3) |  |
|  |  |  | Yes | 43 (10.8) | 112 (4.7) | **<0.001** |
| **Symptoms at admission** |  |  |  |  |  |  |
| Edema | 2793 | 0 (0) | No | 258 (65.0) | 1660 (69.3) |  |
|  |  |  | Yes | 139 (35.0) | 736 (30.7) | **0.087** |
| Chest pain | 2793 | 0 (0) | No | 370 (93.2) | 2337 (97.5) |  |
|  |  |  | Yes | 27 (6.8) | 59 (2.5) | **<0.001** |
| Dyspnea | 2793 | 0 (0) | No | 350 (88.2) | 2331 (97.3) |  |
|  |  |  | Yes | 47 (11.8) | 65 (2.7) | **<0.001** |
| Nausea and vomiting | 2793 | 0 (0) | No | 378 (95.2) | 2336 (97.5) |  |
|  |  |  | Yes | 19 (4.8) | 60 (2.5) | **0.011** |
| Dizziness and headache | 2793 | 0 (0) | No | 310 (78.1) | 1979 (82.6) |  |
|  |  |  | Yes | 87 (21.9) | 417 (17.4) | **0.030** |
| Blurred vision | 2793 | 0 (0) | No | 336 (84.6) | 2198 (91.7) |  |
|  |  |  | Yes | 61 (15.4) | 198 (8.3) | **<0.001** |
| Itchy skin | 2793 | 0 (0) | No | 364 (91.7) | 2206 (92.1) |  |
|  |  |  | Yes | 33 (8.3) | 190 (7.9) | 0.80 |
| **Physical and laboratory tests at admission** | | | |  |  |  |
| Systolic blood pressure | 2774 | 19 (0.7) | — | 156.3 (27.4)^1^ | 149 (23.4) ^1^ | **<0.001** |
| Diastolic blood pressure | 2771 | 22 (0.8) | — | 98.9 (19.0) ^1^ | 94.6 (16.1) ^1^ | **<0.001** |
| Platelet count^3^ | 2769 | 24 (0.9) | — | 4.8 (0.7) ^1^ | 5.0 (0.4) ^1^ | **<0.001** |
| Fibrinogen^3^ | 2721 | 72 (2.6) | — | 5.9 (0.5) ^1^ | 6.0 (0.3) ^1^ | **<0.001** |
| Alanine transferase^3^ | 2677 | 116 (4.2) | — | 3.7 (1.1) ^1^ | 3.5 (1.0) ^1^ | **<0.001** |
| Aspartate transferase^3^ | 2677 | 116 (4.2) | — | 4.1 (0.9) ^1^ | 3.7 (0.8) ^1^ | **<0.001** |
| Total bilirubin^3^ | 2674 | 119 (4.3) | — | 2.5 (0.7) ^1^ | 2.2 (0.5) ^1^ | **<0.001** |
| Urea nitrogen^3^ | 2616 | 177 (6.3) | — | 1.7 (0.6) ^1^ | 1.4 (0.4) ^1^ | **<0.001** |
| Creatinine^3^ | 2635 | 158 (5.7) | — | 4.4 (0.6) ^1^ | 4.1 (0.4) ^1^ | **<0.001** |
| Urine protein | 2488 | 305 (10.9) | — | 4 (1.1) | 79 (3.7) |  |
|  |  |  | ＋ | 81 (21.8) | 721 (34.1) |  |
|  |  |  | ＋＋ | 71 (19.1) | 455 (21.5) |  |
|  |  |  | ＋＋＋ | 92 (24.8) | 418 (19.7) |  |
|  |  |  | ＋＋＋＋ | 123 (33.2) | 444 (21.0) | **<0.001** |

**^1^** Mean (standard deviation); ^2^ Median (interquartile range); ^3^ Logarithmic transformation. BMI: body mass index; ART: assisted reproductive technology; HBsAg: hepatitis B virus surface antigen; GDM: gestational diabetes mellitus. IDA: iron deficiency anemia.

**Table S2 Predictors selected by multivariable logistic regression using imputed data set (*p*-value < 0.5)**

| **Candidate predictors** | **Odds Ratio, (OR, 95%CI)** | ***p-*value** |
| --- | --- | --- |
| Maternal age | 0.98 (0.95-1.01) | 0.13 |
| Pre-pregnancy BMI | 0.98 (0.93-1.02) | 0.31 |
| Rural residents | 0.86 (0.66-1.13) | 0.29 |
| Gestational week at delivery | 0.96 (0.92-0.99) | **0.016** |
| Multipara | 1.25 (0.89-1.73) | 0.19 |
| Use of ART | 0.87 (0.48-1.59) | 0.66 |
| Placenta previa | 2.60 (1.64-4.12) | **＜0.001** |
| Diabetes mellitus | 0.81 (0.35-1.84) | 0.61 |
| Gestational diabetes mellitus | 0.90 (0.59-1.37) | 0.62 |
| HBsAg positivity | 1.74 (1.13-2.67) | **0.012** |
| Cardiac diseases | 3.13 (1.63-6.00) | ＜0.001 |
| IDA | 2.80 (1.98-3.95) | ＜0.001 |
| Chronic nephritis | 1.11 (0.52-2.40) | 0.79 |
| Immune system diseases | 1.94 (0.88-4.31) | 0.10 |
| Fatty liver | 2.55 (0.55-11.83) | 0.23 |
| Hypoproteinemia | 1.41 (0.89-2.23) | 0.15 |
| Edema | 0.82 (0.62-1.10) | 0.18 |
| Chest pain | 1.19 (0.59-2.40) | 0.63 |
| Dyspnea | 3.30 (1.90-5.76) | **＜0.001** |
| Nausea and vomiting | 0.84 (0.40-1.76) | 0.64 |
| Dizziness and headache | 0.87 (0.61-1.26) | 0.47 |
| Blurred vision | 1.28 (0.84-1.96) | 0.25 |
| Systolic blood pressure | 1.01 (1.00-1.01) | **0.032** |
| Hemoglobin^1^ | 0.07 (0.03-0.13) | **＜0.001** |
| Platelet count^1^ | 0.47 (0.36-0.61) | **＜0.001** |
| Fibrinogen^1^ | 0.37 (0.25-0.56) | **＜0.001** |
| Aspartate transferase^1^ | 1.24 (1.04-1.47) | **0.014** |
| Total bilirubin^1^ | 1.50 (1.16-1.94) | **0.002** |
| Urea nitrogen^1^ | 1.08 (0.73-1.60) | 0.71 |
| Creatinine^1^ | 4.34 (2.80-6.74) | **＜0.001** |
| Urine protein |  |  |
| + | 2.49 (0.74-8.31) | 0.14 |
| ++ | 2.48 (0.74-8.34) | 0.14 |
| +++ | 3.47 (1.03-11.70) | **0.045** |
| ++++ | 3.66 (1.09-12.31) | **0.036** |

^1^ Logarithmic transformation. BMI: body mass index; ART: assisted reproductive technology; IDA: iron deficiency anemia.

**Table S3 Internal validation of Model 2**

| **Variables** | **Model 2**  **OR (95%CI)** |
| --- | --- |
| Constant | 3.43e+14 |
| Gestational week | 0.95 (0.92-0.99) ^**^ |
| Placenta previa | 2.81 (1.75-4.51) ^***^ |
| HBsAg positivity | 1.99 (1.28-3.10) ^**^ |
| Cardiac diseases | 3.70 (1.94-7.06) ^***^ |
| IDA | 2.89 (2.02-4.11) ^***^ |
| Dyspnea | 2.93 (1.73-4.97) ^***^ |
| Systolic blood pressure | 1.01 (1.00-1.01) ^*^ |
| Platelet count^1^ | 6.29e-06 (2.27e-07-0.0002) _***_ |
| Fibrinogen^1^ | 0.38 (0.26-0.56) ^***^ |
| Aspartate transferase^1^ | 1.22 (1.02-1.45) ^*^ |
| Total bilirubin^1^ | 1.48 (1.13-1.93) ^**^ |
| Creatinine^1^ | 0.61 (0.11-3.36) |
| Urine protein |  |
| + | 2.58 (0.77-8.60) |
| ++ | 2.52 (0.75-8.50) |
| +++ | 3.45 (1.03-11.61) ^*^ |
| ++++ | 3.82 (1.15-12.71) ^*^ |
| Nonlinear term of platelet^1^ | 2.30 (1.45-3.63) ^***^ |
| Platelet×Creatinine^1^ | 1.09 (1.02-1.17) ^*^ |

^1^ Logarithmic transformation. OR: odds ratio; CI: confidence interval; IDA: iron deficiency anemia. ^*^*P*＜0.05, ^**^ *P*＜0.01, ^***^*P*＜0.001

**Table S4 The model performance in sensitive analysis**

| **Performance** | **Indicator** | **Model 1** | **Model 2** |
| --- | --- | --- | --- |
| **Overall Performance** | Pseudo R^2^ | 0.2457 | 0.2912 |
|  | χ2 statistics | 362.00  （*p*＜0.001） | 429.05  （*p*＜0.001） |
|  | Pearson χ2 | 2265.83  （*p*=0.3249） | 2098.33  （*p*=0.9804） |
|  | Akaike information criterion (AIC) | 0.508 | 0.480 |
| **Discrimination** | Area under ROC curve（95% CI） | 83.13%  （80.12%-86.13%） | 84.91%  （82.17%-87.65%） |
|  | Cut-off | 0.14 | 0.14 |
|  | Sensitivity | 65.20% | 66.08% |
|  | Specificity | 86.71% | 87.25% |
|  | Accuracy | 84.54% | 85.12% |
|  | Positive predictive value | 35.41% | 36.67% |
|  | Negative predictive value | 95.71% | 95.84% |
|  | Discrimination slope | 0.2320 | 0.2812 |
| **Calibration** | Hosmer-Lemeshow goodness-of- fit $\chi^{2}$ | 5.87  （*p*=0.6619） | 7.49  （*p*=0.4844） |

ROC: receiver operating characteristics curve; CI: confidence interval.
